# Unraveling patients’ readiness in advance care planning conversations: a qualitative study as part of the ACTION Study

**DOI:** 10.1007/s00520-020-05799-x

**Published:** 2020-10-01

**Authors:** M. Zwakman, M. M. Milota, A. van der Heide, L. J. Jabbarian, I. J. Korfage, J. A. C. Rietjens, J. J. M. van Delden, M. C. Kars

**Affiliations:** 1 Julius Center for Health Sciences and Primary Care, University Medical Center Utrecht, P.O. Box 85500, 3508 GA Utrecht, the Netherlands; 2 Department of Public Health, Erasmus MC, University Medical Center, Rotterdam, the Netherlands

**Keywords:** Advance care planning, Palliative care, Advance directives, Medical oncology, Health communication

## Abstract

**Purpose:**

Patients’ readiness for advance care planning (ACP) is often considered a prerequisite for starting ACP conversations. Healthcare professionals’ uncertainty about patients’ readiness hampers the uptake of ACP in clinical practice. This study aims To determine how patients’ readiness is expressed and develops throughout an ACP conversation.

**Methods:**

A qualitative sub-study into the ACTION ACP conversations collected as part of the international Phase III multicenter cluster-randomized clinical trial. A purposeful sample was taken of ACP conversations of patients with advanced lung or colorectal cancer who participated in the ACTION study between May 2015 and December 2018 (*n* = 15). A content analysis of the ACP conversations was conducted.

**Results:**

All patients (*n* = 15) expressed both signs of not being ready and of being ready. Signs of being ready included anticipating possible future scenarios or demonstrating an understanding of one’s disease. Signs of not being ready included limiting one’s perspective to the here and now or indicating a preference not to talk about an ACP topic. Signs of not being ready occurred more often when future-oriented topics were discussed. Despite showing signs of not being ready, patients were able to continue the conversation when a new topic was introduced.

**Conclusion:**

Healthcare professionals should be aware that patients do not have to be ready for all ACP topics to be able to participate in an ACP conversation. They should be sensitive to signs of not being ready and develop the ability to adapt the conversation accordingly.

**Electronic supplementary material:**

The online version of this article (10.1007/s00520-020-05799-x) contains supplementary material, which is available to authorized users.

## Introduction

In spite of evidence that advance care planning (ACP) conversations can increase both the quality of care and overall satisfaction of patients with advanced cancer [1–3], the uptake of ACP in clinical practice has remained low [4–7]. Both healthcare professionals (HCP) and patients have indicated that readiness constitutes a significant barrier to initiating these ACP conversations [8–13].

A primary goal of ACP is to help ensure that patients’ medical care and treatment aligns with their personal values, goals, and preferences, especially should they become unable to articulate these preferences themselves [14]. For patients with advanced cancer, an ACP conversation can serve as a valuable opportunity to discuss and refine their wishes and preferences before the onset of progressive and functional decline. An ACP conversation generally consists of four phases: preparation, initiation, exploration, and action. During the core phase, *exploration*, patients are encouraged to share their thoughts about various end-of-life topics [15]; often, they are also invited to discuss topics related to various aspects of their lives including their physical health, psychological, and social wellbeing [16].

Due to the sensitive nature of these topics, both healthcare providers and cancer patients consider readiness for ACP an important factor when deciding whether or not to engage in an ACP conversation. Some studies have defined readiness as being prepared for action [17] or being willing to engage in a discussion about one’s values and wishes with one’s family and HCP [16, 18, 19]. Studies of ACP based on these definitions often consider readiness as a *prerequisite* for a conversation. These studies focus on a patient’s state-of-mind prior to the start of the ACP conversation [14, 16] and consider readiness to be a predictor of a patient’s willingness to engage in an ACP conversation or ACP-related activites [18–21], an indicator for HCPs as to when they should initiate an ACP conversation [22], or an essential precondition for a patient to experience an added value of ACP [14, 16, 23]. Yet readiness has also been defined as a *process outcome* of successful ACP [24], and patients have reported that the ACP conversation itself can have a positive impact on their readiness [25].

Until now, the literature has shed little light on the manifestations of patients’ readiness *during* an ACP conversation. This means that we have very little practical knowledge about how patients respond to individual topics brought up during an ACP conversation or how their state of readiness might shift or change during the course of the conversation [26]. Therefore, this study aims to gain more insight into how signs of (not) being ready become manifest and the role that readiness plays in advanced cancer patients’ discussions of ACP topics throughout a conversation.

## Methods

### Research design

We conducted a secondary analysis of ACTION ACP conversation recordings. An inductive qualitative content analysis of ACP conversations was done in order to better understand how patients responded to the topics being addressed, and ultimately to arrive at a better understanding of the manifestations of readiness for ACP in these conversations [27, 28]. The study data were thematically analyzed. This study is embedded in the ACTION trial (ISRCTN63110516), Phase III multicenter cluster-randomized clinical trial designed to evaluate the ACTION Respecting Choices (RC) ACP intervention in six European countries (Belgium, Denmark, Italy, the Netherlands, Slovenia, and the UK) [29]. Patients with advanced lung or colorectal cancer were recruited to participate in the ACTION trial between May 2015 and December 2018 [29].

### Sampling and data collection

**Fig. 1:**
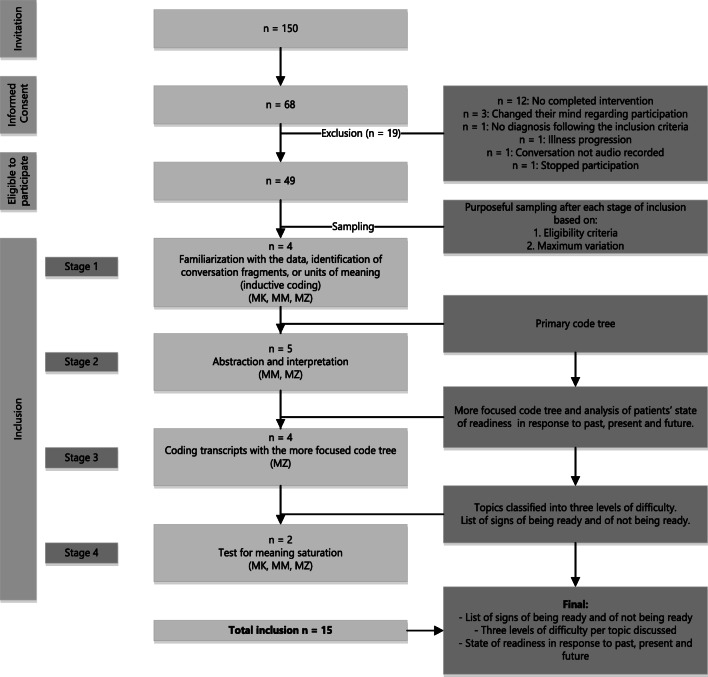
Inclusion, sampling, and data analysis

For this sub-study, we purposively sampled ACTION RC ACP conversations of patients who completed the ACTION RC ACP conversations in both the qualitative and quantitative parts of the ACTION study at one of the participating Dutch intervention sites. A total of 150 patients were invited to participate in the intervention arm of the ACTION study. Sixty-one patients (21 colorectal patients and 40 lung patients) participated in the study (Fig. 1). ACP conversations were eligible for this study when (1) the facilitator involved had already conducted at least three ACTION RC ACP conversations and (2) the ACTION RC ACP conversation was fully completed. The conversations were led by a trained facilitator either at the patient’s home or in the hospital where they were being treated. Facilitators used a structured conversation guide consisting of open-ended questions on ACP topics as well as scripted explanations of key concepts [29]. Table 1 includes a list of the topics discussed and an example of a corresponding question per topic (see Supplementary Material 1 for more information about the intervention). We considered an ACP conversation to be completed when all scripted discussion guide topics had been broached. This could require one or two ACP conversations depending on the patients’ wishes and the presence of a so-called personal representative—a person appointed by the patient to express their preferences should they be unable to do so themselves. An ACTION RC ACP conversation lasted an average of 1 h and 25 min. To increase our understanding of readiness in all its iterations, maximum variation was sought while sampling with respect to patients’ underlying illness, treatment, and facilitators, as is common in qualitative studies [30].

**Table 1:** Topics ACTION RC ACP conversation

Topic	Sample question
1. Understanding of role of the PR	What do you understand about the role of the Personal representative?
2. Patient’s and PR’s understanding of ACP	Have you done any advance care planning before?
3. Understanding of illness	Tell me what you understand about your illness
4. Complications	What do you understand about the possible complications of your illness and what might happen in the future?
5. Experiences	What did you learn from that experience (experiences with family or friends who became ill or injured and were not able to communicate)?
6. “Living well”	What does living well mean to you?
7. Worries and fears	Do you have worries about your illness or medical care? If so, what worries do you have?
8. Possible personal, cultural, religious, or spiritual beliefs	Do you have any personal or cultural beliefs that might influence your preferences for future care and treatment?
9. Patient’s hopes for current medical plan of care (part 1) 10. Patient’s hopes for current medical plan of care (part 2)	What do you hope for with your current medical plan of care? I understand these hopes. If all these hopes do not come true, what else would you hope for?
11. Help making an informed decision regarding CPR	What do you understand about resuscitation?
12. Discuss goals, values, and preferences for future complications	Tell me in your own words what you understand about this option (Selective Treatment plus Comfort-Focused Care)?
13. Preferences relating to the final place of care	Do you have preferences relating to the final place of your care?

Data about the patients’ characteristics were collected from their medical files. The location of the ACP conversation and the number of conversations per patient were extracted from the facilitators’ reports of the ACTION RC ACP conversations. The included conversations were transcribed verbatim by a professional transcription service and checked for reliability (MZ) prior to the data analysis.

### Data analysis

The data selection and analysis occurred inductively and iteratively [31] by means of constant comparison [32]. The software program NVivo11 supported the data analysis. The members of the analysis team are experienced qualitative researchers; two of them (MK, MZ) have a background in nursing. From the conversations of 49 participants that met our eligibility criteria, we first sampled four conversations of patients with different diseases and facilitators in order to familiarize ourselves with the material. After reading the transcribed conversations as a whole, three members of the analysis team (MK, MM, MZ) independently reread the four transcripts in order to identify conversation fragments, or units of meaning [28], we thought signaled signs of being ready and of not being ready for ACP. At this phase of the analysis, we tried to focus on manifest rather than latent content, keeping close to the data and working with a low level of abstraction [27, 28]. We then started grouping these fragments into descriptive categories, resulting in our first code tree. Next, we sampled five more conversations varying in patient gender, levels of experience of the facilitators, and stage of illness. MM and MZ independently coded these transcripts. These independent coding results were compared and discussed during weekly meetings until consensus about the codes, provisional categories, and interpretations was reached. MZ then sampled and coded four more transcripts.

After the first stage of coding, we began a process of abstraction and interpretation [27]. We noticed that facing the past, present, or future played a role in patients’ readiness. As a result of these insights, we created a more focused code tree and fine-grained analysis of patients’ state of readiness for all 15 conversations in response to past, present, and future situations. Again, MK, MM, and MZ discussed differences in their interpretation during weekly meetings until consensus was reached. Using our analyses of variations in readiness in the preceding two stages as a guide, we then categorized the topics discussed during the ACTION RC ACP conversations into three levels of difficulty. Finally, MK, MM, and MZ tested for meaning saturation [33] by sampling and coding two new conversations with the most experienced facilitators and using the final list of signs of being ready and signs of not being ready.

### Ethical considerations

Ethical approval for the ACTION trial, including the qualitative work package, was obtained from the Research Ethics Committee (REC) of Erasmus MC, University Medical Center Rotterdam (14-560/C). Written informed consent was obtained from all participating patients. Verbal informed consent was obtained and recorded from the relatives present at the ACP conversation. To ensure confidentiality, all transcripts were coded and any identifying information was removed.

## Results

Fifteen of the 49 eligible completed ACP conversations were included for analysis (32%). Table 2 includes an overview of the patient and facilitator characteristics.

**Table 2:** Background characteristics

	*N* (%)	
*N* patients	15 (100)	
Male	7 (46.7)	
Age	64.8 years (range 51–79 years of age)	
Marital status		
Married/civil partnership	14 (93.3)	
Living with a spouse/partner	15 (100)	
Living in a private household	15 (100)	
Having children, yes	13 (86.7)	
Number of children living at home	0	
Being religious	8 (53.3)	
WHO		
0	4 (16.7)	
1	11 (73.3)	
Diagnosis		
Lung cancer (stage III or IV)	10 (66.7)	
Colorectal cancer (stage IV or metachronous metastases)	5 (33.3)	
Current treatment*		
Chemotherapy	6	
Radiation therapy	5	
Immunotherapy	5	
Targeted therapy	2	
Current cancer-directed treatment		
Palliative	12 (80)	
Curative	1 (6.7)	
Unknown	2 (13.3)	
Facilitators	**Involved	**Not involved
Facilitator 1 lung (female)	4	
Facilitator 2 lung (female)		6
Facilitator 1 colorectal (female)		3
Facilitator 2 colorectal (male)		2

*Some patients received more than one treatment at the same time

**Involved or not involved in regular care for interviewed patient

### Unraveling patients’ readiness

From the conversations, we identified both signs of not being ready and signs of being ready.

#### Signs of not being ready

Signs of not being ready were found in all conversations (Table 3). Patients signaled their unreadiness by expressing a reluctance to consider aspects of ACP, by minimizing the seriousness of their illness and condition, and by steering away from talking about the personal consequences of their illness progression. These signs indicated that a patient’s inability or unwillingness to talk about certain ACP topics was not just a reflection of the patient’s state of mind at that moment in the conversation. Many of the signs of not being ready also revealed that a patient was delaying or avoiding having to think about his/her own deterioration of health or death.

**Table 3:** Signs that a patient is not ready for aspects of ACP

Code	Description and interpretation	Sample of text fragment
Keeping things out of sight	When asked to consider future scenarios and articulate a clear preference about end-of-life care, the patient avoids taking a definitive stance by either stressing the unpredictability of the future or postponing a decision until an unspecified later moment. This may help a patient maintain a sense of control over the current situation, manage worries and anxieties, or prevent unnecessary worrying. *The patient:* • Avoids talking about the end of life • Avoids what he/she considers an emotionally painful topic • Describes a desire to preserve a sense of uncertainty • Delays having to make difficult decisions or indicate a preference • Keeps the possibility open for an improvement or cure	I: I understand the hope that you’ve just mentioned, but what if this hope can’t be realized, that you reach a point where you decide to stop with the treatments, what would you hope for then? R: I don’t dare think about that right now. I: That’s too far away, eh. R: We’re pushing that away with a big bulldozer. *(Pat 2. 67 years of age)*
Putting a stop to the discussion	The patient actively puts a stop to the exploration of the topic by declining the facilitator’s offer to provide more information or by simply refusing to discuss the topic any further. This may help the patient maintain a feeling of control over his/her life and emotions or protect him/herself from unnecessary or emotionally painful information. *The patient:* • Avoids talking about physical deterioration or end of life • Indicates a desire to avoid unnecessary worrying • Delays having to learn about negative outcomes • Employs curative or “fighting” rhetoric	I: Then is the question what do you know about the possible complications of your illness, what in the future may possibly happen. Do you know anything about this? R: Now, I understood that to mean that if your liver stops working you poison yourself. For the rest I don’t want to know how sick I may eventually feel or which functions I may lose, all the things I won’t be able to do anymore. Because that is one of my fears, that I’ll only be lying in bed waiting until I die. R2: That’s not for you. R: No, I need to be able to go outside and I need to be able to do things (laughs). I: Yes, in that respect it could be helpful if Doctor K could talk to you [about the complications] so that you know whether or not you have to adjust your expectations. R: Yes, at some point. I: At some point. R:For me it’s not necessary yet. *(Pat 3. Female, 60 years of age)*
Limiting one’s perspective to the here and now	The patient refuses to consider the future when asked to do so in the ACTION ACP RC script and chooses instead to remain present-centered. This may help a patient protect him/herself from negative or sensitive information. *The patient:* • Delays having to think about him/herself in a deteriorated condition • Employs curative or “fighting” rhetoric • Tries to stay positive	I: Let’s say that your wife has to make the decision at a certain moment about whether or not to resuscitate. What would your advice for her be? R: As it is now, yes [resuscitate]. I: No, but if you can’t speak anymore, eh? That would mean something has happened. R: Yes, but I’m pretty good now, so I would definitely say try to resuscitate me. *(Pat 2. Male, 67 years of age)*
Minimizing the seriousness or significance of one’s symptoms	When asked about the progression of the illness, the patient avoids having to consider the seriousness of the situation and chooses instead to focus on the positive aspects of the treatment or a small improvement in health, downplay the symptom burden, or mention unrelated illness symptoms. This may help the patient maintain a sense of control over the situation. It may also indicate an effort to suppress, fragment, or avoid signals of deterioration by focusing instead on details that can be managed or easily explained. *The patient:* • Actively steers the conversation in a positive direction • Avoids talking about physical deterioration or death • Employs curative or “fighting” rhetoric • Tries to stay positive	I: Has your illness changed in the last months? R: No, I have to say with the deteriorated liver function that I really felt a new dip and that you immediately also think: I’m more tired, is my condition going to get worse, and is there something wrong, do I have more pain now? And I actually have that every time for 1 or 2 days after I get bad news, or if it sounds like bad news to me, and then it gets better. I switch that button again, then I think: how bad is it if you can’t eat candy anymore and have to drink more water? You just have to keep swallowing the hormone pills, period. And these are the consequences, deal with it. *(Pat 7. Female, 52 years of age)*
Distancing oneself from the topic being discussed	When discussing various ramifications of a deterioration in quality of life or death, the patient can provide an answer but distances him/herself from the topic. This may be a strategy for maintaining control over his/her emotions. It could make it easier to articulate his/her stance. It may also help a patient make it seem like the decision or stance is not merely his/her own. *The patient:* • Switches from the first- to second- or third-person perspective • Makes his/her observations more general and less personal	R: Yes, I’ll talk about it with her [HCP] again, I’ll say: now explain to me what is your image, idea, about when I will die, and what are the symptoms that that will go along with that. And what is for me acceptable, what isn’t? Now there is a limit [to what is acceptable], and that I need to get clear. […] R: Yes, because the limit may change, every time different. I think that’s how it is with a lot of people. I: Yes. It is difficult to establish a limit, because maybe it doesn’t work that way. R: No, you can’t just determine the limit. You only realize it when you experience it, then you say: it’s finished. *(pat 12. Male, 71 years of age)*

#### Signs of being ready

Conversely, the willingness and ability to discuss an ACP topic or to consider the personal relevance and impact of an ACP topic constituted important indications that a patient was ready for that ACP topic. Table 4 lists the signs of being ready identified. Although patients indicated their readiness to discuss an ACP topic in a variety of ways, each sign of readiness essentially revealed that a patient could face and talk about an aspect of ACP and could link his/her thoughts to future scenarios related to the end of life.

**Table 4:** Signs that a patient is ready for aspects of ACP

Code	Description and interpretation	Sample of text fragment
Indicating basic readiness	The patient shows a willingness and ability to discuss an ACP topic and links the answer to his/her own experiences or personal situation. *The patient:* • Makes links between the ACP topic and his/her personal situation • Answers the ACP question seriously	I: Do you have any worries about your illness or treatment? R: No. Yes, you are going to die, but you knew that already. Even if I hadn’t gotten sick. Look, I’m 73, I have nothing to complain about. *(Pat 8. Male, 73 years of age)*
Spontaneously mentioning ACP-related topics independently of the script prompts	The patient independently brings up an ACP-related topic and indicates that he/she has previously considered the topic and is therefore ready to discuss this topic with the facilitator. *The patient:* • Has a strong preference or wish regarding a certain aspect of ACP • Has considered possible steps that will need to be taken in the future • Has reflected upon his/her present situation • Has already made decisions regarding his/her future care • Has proactively arranged for his/her future care and discussed this with his/her HCP	I: Are there other personal beliefs that matter in regards to your future care and treatment? R: No, well in regards to resuscitation, then of course it would be: do not resuscitate. *(Pat 7. Female, 52 years of age)*
Learning from past illness experiences	When considering a previous personal illness experience or that of a family or friend, the patient can not only describe the experience, but can also draw lessons from the experience. This may indicate an ability to link the past to his/her present state and stance. It may also signal that a patient has thought about the significance and meaning of another person’s suffering and death and can transfer it to his/her own life and situation *The patient:* • Relates a previous experience with illness to his/her own thoughts, feelings, and preferences • Uses an example of an illness experience to help formulate and articulate his/her own values, goals, and preferences	R2: So that means that you don’t endlessly treat, treat, treat. R: Because that would be treatment for treatment’s sake. R2: If the results are dubious, and the chance of a positive result are really small, and that it has a negative influence on the quality of life, then you would choose not to be treated and to enjoy the last few months. We experienced this with friends in France, where the situation is different, the doctor-patient interaction, too. And there they kept treating and treating, and we said afterwards, he would have been a lot happier if he had died 6 months earlier, then he would have been happier than with the year and a half he had to endure. R: Yes. R2. So that’s the difference. […] R: Yes, if you keep treating for the sake of it, or if you are treating to reduce symptoms, even if the man is getting worse and worse. No. *(Pat 4. Female, 67 years of age)*
Demonstrating an understanding of one’s diagnosis and current state of health	The patient can clearly and realistically articulate a view of his/her situation and can describe what medical information means to him/her personally. *The patient:* • Attempts to describes the situation as it is • Describes why and how information related to his/her illness is personally significant • Provides a nuanced description of the diagnosis and current state of health • Provides a realistic explanation for changes in his/her symptom burden	I: What do you know about your illness? R: I know that I have stomach cancer, that is the primary cancer, and it’s metastasized to my peritoneum and my liver. And that it can’t be cured because the tumors in the liver, they’re located on inoperable spots, they’re tiny. On the CT scan you can’t even see all of them, but you can on the MRI. Nevertheless, the surgeons can’t find them, so it’s inoperable. And because the liver is inoperable it doesn’t make sense to operate on the other tumors. It makes more sense to talk about the quality of life you have, according to Doctor X, to try to keep it under control for as long as possible. *(Pat 10. Female, 56 years of age)*
Demonstrating and understanding of one’s disease and prognosis	The patient demonstrates a clear understanding of the seriousness of the situation and what this may entail in the future. This may indicate that a patient is not avoiding the prospect of a deterioration in quality of life and death. *The patient:* • Indicates that he/she has considered that his/her illness may be incurable • Can describe what a future deterioration of health might entail	I: You say that this is the third time in two years [that you’ve had lung cancer]. You had it earlier and it has returned. R: Yes, limited. A half lung has been removed, and a half year later there were metastases in the lung and chest glands. And now a year later the cancer is in both lungs and the liver. So that means end of story. It’s finished. *(Pat 13. Female, 61 years of age)*
Considering the topic from various sides	The patient demonstrates that he/she can weigh the pros and cons of various decisions, consider the last phase of life from different angles or perspectives, and can reflect upon a previous experience with illness by considering various actors and effects. This may indicate that a patient can see his/her own illness in a broader context and is willing and capable of linking the topic to his/her emotions. *The patient:* • Can describe what a future deterioration of health might entail • Reflects upon his/her own situation or experiences • Has previously thought about ACP-related topics • Is willing to ask for more information regarding his/her situation and possibilities for his/her future care and treatment • Reflects upon his/her good and bad feelings or worries in his/her daily life	[In regard to choosing complete treatment or comfort treatment] R: I would choose for comfort. I think that comfort is the priority for me. It’s not like I want to live a few more months at all costs, no. I: No. R: But if it yields something, if it yields real quality. If I have a bladder infection and it’s simple to treat with antibiotics, great. But if they say, now it’s in your lungs, and you know that treating a lung infection would mean that you would then have to remain on an oxygen machine, then no. *(Pat 2. Male, 67 years of age)*
Anticipating possible future scenarios	The patient can face and talk about end-of-life topics such as future complications, reanimation, and place of final care and has thought about and can anticipate a deterioration in quality of life and death. This may indicate that a patient is capable of thinking and talking about death. *The patient:* • Has actively considered the last phase of life • Describes a pragmatic or realistic view of the future • Is prepared to consider the steps that may need to be taken to ensure that his/her goals and preferences are honored • Is sensitive to his/her own future needs as his/her disease progresses • Can articulate his/her emotions regarding a future deterioration of health • Actively searches for a realistic description and understanding of his/her future symptoms	R: I’ve made it completely clear to my children that I don’t want to live in a vegetative state in bed waiting until I stop breathing, that there may be a moment when euthanasia becomes a desired option. And my GP told me that this wouldn’t be a problem in my case, it’s clear my suffering is hopeless and unbearable. When I talked with him about the things I might be scared about, things that might happen, he told me that I didn’t need to be scared because he would sedate me. We talked about that sort of things. *(Pat 6. Female, 64 years of age)*
Accepting one’s disease and deterioration of health	The patient demonstrates an acceptance of the seriousness of the disease and demonstrates that he/she has previously thought about and come to terms with a deterioration of health and death. *The patient:* • Is willing and able to talk about his/her end of life as a given fact • Actively reflects upon his/her life and relates these reflections to the topic being discussed • Spontaneously anticipates and mentions his/her own death • Describes the gravity of the situation	I: What does a good life mean for you, what, for instance, does a good day look like to you? R: You mean right now, not in the past? I: I would hope that your answers would be similar, but… R: Now, the answers are quite far apart, depending on what you make of it. A good life is what we’ve done, what I’ve done, at the moment that you realize that it’s going to end, then you look back at your life. *(Pat 12. Male, 71 years of age)*

#### Shifts in readiness within the conversation

Our analysis of the transcripts showed that patients could display *both* signs of not being ready and of being ready for ACP within one conversation and even within one topic. In fact, patients’ state of readiness could shift per sentence. We also noticed that if a patient had difficulty with one topic, this did not imply he/she would also have difficulty discussing the subsequent topic in the script. To illustrate, Table 5 provides a summary of the ACP conversations of two patients. For example, patient 9, who was unwilling or unable to talk about topics such as his diagnosis and potential future complications, and who openly struggled emotionally at multiple points throughout the conversation, could nevertheless clearly and resolutely articulate his preferences regarding resuscitation and his final place of care.

**Table 5:** Samples of shifts in readiness during the ACTION RC ACP conversation

Topic	Patient 2 (male, 67 years of age, PR is his wife)		Patient 9 (male, 77 years of age, PR is his wife)	
1. The role of the PR	*Ready:* Has read the folder about the role of the PR prior to the conversation, presumes, and expects that his wife will carry out the tasks required by a PR.		*Ready:* PR can describe her role and function as a representative if her husband can no longer speak for himself, namely that she will communicate his preferences and decisions. Patient is confident she will be able to do this even in difficult situations.	
2. Practice and understanding of ACP	*Not ready:* No previous experience with or knowledge of ACP, patient, and PR both admit that patient is “not a talker.”		*Ready:* Has thought about resuscitation (does not want CPR); has discussed this preference with HCP.	*Not ready:* Has purposefully delayed talking about other preferences until the future, or when it is necessary.
3. Understanding of illness	*Ready:* Can state diagnosis: colon cancer with metastasis in the liver.	*Not ready:* Stresses the positive aspects of treatment, minimizes the side effects of chemotherapy to pain in his fingers.	*Ready:* Can state diagnosis: lung cancer with four metastases.	*Not ready:* Knows very little about illness (i.e., location of lung tumor, purpose of chemotherapy).
4. Complications	*Not ready:* Does not want to think about complications, prefers to delay such a conversation until it’s necessary, to avoid worrying.		*Not ready:* Clearly states that he does not know about complications, does not want information about complications, and does not want to think about his own death.	
5. Experiences with family or friends who became ill	*Unclear:* Patient answers “no” to the question, so PR tells about an illness experience in the family.	*Ready:* At a later point in the conversation, the patient spontaneously describes his own previous illness experience 40 years prior and links his previous coping strategy to his current coping strategy.	*Ready:* Can tell about illness experience of family member.	*Not ready:* Cannot think of any links to his own situation.
6. ‘Living well’	*Ready:* Describes what living well means to him: living without physical hindrances or constraints.	*Not ready:* When asked to consider what “living well” would mean in the future with a deteriorated state of health, patient says he can’t think of or give an answer. PR says this is because he’s not the type to think ahead.	*Ready:* Describes what living well means to him: to wake up healthy, to be together with his family, children, and grandchildren.	
7. Worries about illness or medical care	*Ready:* Admits to be scared that the current treatment will eventually stop being effective.	*Not ready:* Pronominal shift to second-person perspective, wants to keep the future uncertain and “make the best of it.”	*Not ready:* Only wants to talk about positive aspects of treatment, does not want to think about future. Additional information: Becomes emotional when answering.	
8. Possible personal, cultural, religious, or spiritual beliefs	*Ready:* Patient says that he used to go to church and for that reason could be considered a believer.	*Not ready:* When asked to describe the most important aspects of his belief for him personally, patient responds that he “can’t put it into words.”	*Ready:* Talks about Roman Catholic faith.	*Not ready:* Repeats “I don’t want [to think about] it yet” when conversation turns to topic of his own religious funeral service.
9. Hopes for current medical plan of care	*Ready:* Can clearly articulate his hopes: to stay alive for a long period of time and to be cured.	*Not ready:* Pronominal shift from ‘I’ to ‘we’ when discussing hopes.	*Ready:* Can clearly articulate his hopes: to feel better every day, that the illness will disappear, that he will leave the hospital cured. Additional information: Becomes emotional when answering.	
10. Hope should other hopes go unfulfilled	*Not ready:* Does not want to think or talk about it.		*Ready:* Can answer the question with an alternative hope: that he can live as long as possible.	
11. Preferences for resuscitation	*Ready:* Expresses a clear preference to be reanimated in a current physical state. Anticipates future scenarios and admits that this preference may change. Adds that at a certain point in the future he would rather not be reanimated.		*Ready:* Expresses a clear wish not to be in a vegetative state and has already communicated this wish to his HCP and family. Mentions this preference spontaneously multiple times in the conversation.	
12. Care during final phase of life	*Ready:* Describes various possible scenarios that are related to his own illness.	*Not ready:* Delays making a choice because of uncertainty about the future and the variety of possible scenarios.	*Ready:* Expresses a clear preference for treatment of all future possible complications.	
13. Final place of care	*Ready:* Would like to die at home, but realizes this will depend on the situation. PR adds that she will do her best to honor his wish and names people who can help her care for him if necessary.		*Ready:* Expresses a clear preference to die at home where his wife can help care for him.	*Not ready:* Pronominal shift to third-person when talking about the end of life.
			Additional information: Becomes emotional when answering.	

### Understanding patients’ readiness

Deeper analysis revealed that the level of readiness was most visible in patients’ willingness and ability to face their future illness trajectory while taking the past and present into account. Patients’ level of readiness also hinged on the ability to imagine and face the personal consequences of their illness trajectory, both in the physical and psycho-social spheres.

#### Easy and difficult ACP topics

All patients in this study were able to participate in the ACTION RC ACP conversation, but we found a great variation in their willingness and ability to talk about the distinct topics. The topics discussed during an ACTION RC ACP conversation can broadly be categorized into three levels of difficulty (see Table 6), which correspond to the signs of being ready and of not being ready we identified per topic. In line with the insights presented above, the predominantly easy topics were ones that patients could discuss without linking it to their personal situation—such as the concept of ACP—or topics that facilitated a positive view about their life and illness. The most difficult questions were the ones that explicitly challenged patients to link their responses to their own lives, thoughts, and feelings and to imagine themselves in specific future situations.

**Table 6:** Easy and difficult topics in the ACTION RC ACP conversations

Predominantly easy topics	Somewhat difficult topics	Predominantly difficult topics
• Designation of a personal representative • Previous knowledge or practice of ACP • Earlier experiences with illness in their social or familial circle • Personal definition and description of ‘a good life’ • Hopes (part one of two-part question)	• Religious or spiritual beliefs • Diagnosis • Preferences regarding resuscitation • Goals of future care (complete treatment or comfort-oriented treatment) • Final place of care	• Knowledge of potential future complications • Worries and questions about illness • Hope should other hopes go unfulfilled (part two of two-part question)

Listed in the order they appear in the ACTION RC ACP script

#### The role of reflection and prospection in a patient’s readiness for ACP

The ACTION RC ACP script encouraged patients to reflect upon the past, the present, and the future at certain moments during the conversation. Most patients were ready to say something about the present and were ready to reflect upon the past. But when asked to link past experiences to their present situation or to think about the future—be it possible future complications or preferences regarding end-of-life care—we noticed more diversity in patients’ states of readiness. Many patients delayed or avoided talking about the future or of what could be learned from earlier experiences (see Table 3). And as Table 4 illustrates, those patients who were ready and able to think about the future could consider their own changes in health from the past, the present, and the future position. They demonstrated an informed view of their prognosis and could anticipate future scenarios; some patients could also shift between the past, present, and future spontaneously and independently of the script.

#### Rational versus experiential perspective-taking

We noticed a further differentiation in the manner in which patients articulated their stance toward an ACP topic: via *rational* and *experiential* perspective-taking*.* Most patients took a rational approach and spoke without discernible emotional distress about past- and present-focused topics such as experiences with illness in their social or familial circle or when describing what “a good life” entailed. Most patients could also rationally describe practical matters related to the future, such as funeral arrangements, financial arrangements, or the eventual reallocation of household tasks. While these future matters pertained to them directly, patients almost always discussed these matters in an abstract or generalizing manner. To illustrate, one patient answered the question regarding the completion of advance directives as follows: “my non-resuscitation wishes and the euthanasia form [living will], are signed and are all here [in a folder] and [also] with my doctor” *(Patient 12)*. This patient shared only the technical side of his end-of-life wishes without giving any impression of what they meant to him personally.

Some patients who were ready to discuss an aspect of ACP could also imagine themselves in various situations or consider the significance of a specific topic for them personally. This experiential perspective was most apparent in patients’ answers to the future-oriented ACP prompts. For example, one patient repeatedly stated during a conversation that he did not want to end up in a vegetative state. When asked by the facilitator to expand upon this statement, the patient responded: “As long as my brain still works I think I can deal with a lot of physical burdens. But for me it’s all about brain function. If I don’t recognize people anymore. I think that would be terrible for the people around me, but also for me. That is what I consider a vegetative state” (*Patient 6).* This patient’s utterance indicates that he could imagine himself in a future situation of physical deterioration and suffering. Of the patients who were more ready to face and discuss their future, a sub-selection appeared to be able to imagine the course their illness would likely take and how they personally would react to the impeding changes in health.

### Synthesis of readiness for ACP

Synthesizing our findings, we arrived at the following description of readiness for ACP. It is necessary to note that most patients in our study were *partially* ready for ACP, meaning that they could talk about some, but not all ACP topics.

**Table:** 

*Readiness* for ACP is the *willingness* and *ability* to engage in a discussion about the progression of one’s illness, one’s current physical and/or mental state, and possible future scenarios related to the end of life; one is also optimally ready for ACP when one can both rationally articulate one’s stance toward end-of-life topics, can articulate one’s corresponding emotions, and can imagine oneself in future situations.	

## Discussion

### Main findings

This study of recordings of scripted and facilitator-led ACP conversations in patients with advanced cancer revealed that patients could display both signs of being ready as well as signs of not being ready when discussing ACP topics. We noticed that signs of not being ready and signs of being ready frequently occurred when patients discussed future-oriented topics related to a deterioration of health and the end of life. Patients who were most ready to talk about an ACP topic were able to envision their own future deterioration of health and to describe what this meant to them personally.

We defined readiness not only as a willingness to engage in a discussion about the progression of one’s illness, one’s current physical and/or mental state, and possible future scenarios related to the end of life, but also as an ability. For example, we identified the following levels in patients’ ability to respond to questions about their future: most patients could rationally articulate their stance toward end-of-life topics, some patients could also articulate their corresponding emotions, and some could even imagine themselves in future situations and reflect on what this meant to them. Patients varied in this ability, and this in turn became manifest in signs of not being ready.

Our study revealed that patients do not have to be ready for all elements of ACP to participate in an ACP conversation. When asked about various ACP topics, patients can respond to questions they feel ready to discuss. Exposure to topics that might trigger signs of not being ready can at least make a patient aware of an end-of-life topic, a first step in the circle of awareness, recognition, acknowledgment, and acceptance. This hypothesis is supported by bereavement theory, showing that “‘adaptive’ coping with loss is a dynamic regulatory process of oscillation between loss and restoration stressors, whereby the grieving individual at times confronts, at other times avoids, the different tasks of grieving.” [34] Although this model was developed for bereavement with loss, we suggest it also fits the situation of coping with anticipated loss due to a progressive illness that necessitates facing the end of life. On the one hand, ACP conversations touch upon loss-oriented stressors; they ask participants to consider how they will address or process various aspects of the loss experience itself. Examples include questions pertaining to life-prolonging treatment and questions related to the anticipated loss of independency due to expected physical deterioration. On the other hand, ACP conversations also address restoration-related stressors. This includes questions about how participants maintain a good life or what they do to add value to lives, which also requires efforts and energy [35]. ACP conversations therefore entail more than identifying and sharing values, goals, and preferences. From the perspective of coping with loss, ACP can also have a therapeutic value as it might add to the patient’s preparedness. This is in line with previous studies that the ACP process itself can have a positive influence upon the patient’s readiness [25]. Just as bereaved persons have to adjust to their new reality in the absence of a beloved person, seriously ill patients also have to adjust to a continuously changing or an anticipated “new reality.” An aspect of this adjustment is preparedness. Studies show that preparedness is supported by discussions that include prognoses and future care [36].

In our study, we also found that patients could answer difficult questions even if they were emotionally difficult. Taking these points into consideration, readiness should not be seen as an unequivocal prerequisite for starting an ACP conversation, but rather as a state of mind that fluctuates throughout an ACP conversation. The fact that ACP conversations trigger grief simultaneously serves as an argument for dosing such grief, and for consciously dosing ACP conversations as well [34]. Patients do need moments of respite from dealing with grief stressors as an integral part of adaptive coping. As such, ACP conversations should be clearly announced and planned to allow the patient and family to prepare for such a conversation.

### Strengths and limitations

A strength of this study was that investigator triangulation was applied by including three researchers with different professional backgrounds and expertise in the data analysis team. This lead to in-depth discussions about how to interpret and categorize the signs of being ready and of not being ready. Another strength is that we studied facilitated conversations that were structured by a conversation guide. The facilitators were trained to bring up and to explore all the listed ACP topics. As a consequence, the topics discussed were the same in all conversations, which enabled us to study readiness in relation to a broad range of ACP topics.

However, the fact that we only studied facilitated and structured conversations can also be considered a study limitation. We could not compare these conversations with open-interview ACP conversations conducted by a patient’s physician or nurse, for instance. Another limitation of this study is the patient sampling; the cases we analyzed were predominantly married and were advanced cancer patients receiving palliative care; we had far fewer cases of patients receiving curative cancer treatment. Finally, it should be noted that patients who were willing to participate in the ACTION trial might have self-selected as being receptive to and probably more ready to discuss ACP in general.

### What this study adds

Our study has concrete implications for practice*.* First, while it is important that patients express general readiness by agreeing to participate in an ACP conversation, HCPs should not use the patient’s readiness as the only indicator for whether or not to initiate or postpone an ACP conversation. Readiness can fluctuate and change during the course of the ACP conversation itself. Instead, HCPs should initiate an ACP conversation with the awareness of the patient’s individual needs, signs of being ready and of not being ready, and potential triggers of signs of not being ready. For example, if a patient seems to show a lot of signs of not being ready to discuss certain aspects of ACP, the HCP can adjust the order of the topics or switch between easy and difficult topics. In addition, knowing that patients can alternate in readiness depending on the topic that is being discussed can help HCPs guide the patients through the conversation accordingly. A topic that deserves further exploration is the impact of facilitators’ attitude and communication skills on patients’ readiness to discuss ACP topics.

## Electronic supplementary material

ESM 1 (DOCX 19 kb)

## Data Availability

The datasets generated and/or analyzed during the current study are not publicly available, but are available from the corresponding author on reasonable request. The data is stored at the central drive of the Julius Center for Health Sciences and Primary Care at the UMC Utrecht with restricted access and protected by a firewall. After the project is completed, data will be stored for the long term (15 years) at the central drive of the Julius Center.
