# Supplementary material for: Unraveling patients’ readiness in advance care planning conversations: a qualitative study as part of the ACTION Study
Source: Support Care Cancer. 2020 Oct 1;29(6):2917–29. doi: 10.1007/s00520-020-05799-x (PMC8062377; doi:10.1007/s00520-020-05799-x)
Supplement: Supplementary file 1 — (DOCX 19 kb) [file 520_2020_5799_MOESM1_ESM.docx]

**Supplementary material 1.** The ACTION Respecting Choice Advance Care Planning intervention

| In the ACTION trial, we evaluate the ACTION Respecting Choices (RC) Advance Care Planning (ACP) intervention. The ACTION RC ACP intervention is an adapted and integrated version of the RC ® First Steps and Advanced Steps RC facilitated ACP conversation. The RC facilitated ACP conversation is one component of the more comprehensive RC ACP programme that was developed and implemented in La Crosse, Wisconsin, and also includes health care system redesign; the education of the whole health care team; patient and community engagement; and ongoing management with quality improvement. More details can be found at [www.respectingchoices.org](http://www.respectingchoices.org).  **Translation**  ACTION RC ACP intervention materials were drafted in English and were translated into the languages of the countries participating in the ACTION trial, in close collaboration with the RC programme developers. In this translation process, materials were, where necessary, adapted to local cultural and ethical nuances, whilst not losing the content, structure and integrity of the RC ACP facilitated conversation. In addition, we developed the so-called My Preferences form. The My Preferences form can be used to document the patient’s goals, values, and preferences. Depending on local legal regulations, the My Preferences form can be used as an Advance Directive.  **Education and Certification**  The ACTION RC ACP intervention consists of one or two conversations between the patient and, if he or she wishes, a relative, and a certified facilitator (mostly a nurse). In each country, 4-10 facilitators, in total 39, participated in a two-day RC First and Advanced Steps training programme given by a certified RC teacher. The training programme included role plays, videos demonstrating RC ACP conversations, and one additional day homework assignments. Trained facilitators were certified using competency based criteria. During the ACTION trial, facilitators received regular support and feedback from the RC teacher, based on audio-recorded conversations. Furthermore, facilitators had the opportunity to share their experiences or to discuss difficulties with the RC teachers.  **Main elements of the ACTION RC ACP intervention**  1. ACP CONVERSATION GUIDES  The ACTION RC ACP conversations are structured by the use of conversation guides that include scripted questions, information and the integration of general interview (communication) skills. Based on these guides, facilitators support patients and their relatives in exploring the understanding of their illness, in reflecting on their goals, values and beliefs, and in discussing their preferences for future treatment and care. The intervention also supports patients in identifying specific activities and experiences that may contribute to, or detract from, their quality of life and future care planning.  There are three conversation guides that facilitators select for different situations:   - The blue guide: for the first conversation with the patient and a personal representative (PR). - The green guide: for the first conversation with the patient, but without a PR. - The yellow guide: for a follow-up conversation with the patient and a PR.   These conversation guides include a variety of topics. To start, patients are supported in identifying a PR, who preferably also attends the follow-up ACTION RC ACP conversation. This enables the PR to become familiar with the patient’s views and wishes and encourages an open dialogue between the patient and the PR. Next, the script continues with the following key topics: what is the patient’s understanding of their disease and possible complications, what did the patient learn from previous experiences with family or friends who became ill and were not able to communicate, what are the patients’ beliefs, what are the patients’ fears and worries, what is the patient hoping for, and what is important for the patient to live well. In addition, the patient’s preferences concerning resuscitation, goals of care, and final place of care are discussed. Finally, patients are informed by the facilitator that they can document their preferences for future medical treatment and care in the My Preferences form. Patients are encouraged to discuss their preferences and questions with their attending physician.  2. MY PREFERENCES FORM  The My Preferences form is partly based on the RC Power of Attorney for Healthcare and the Physicians Orders for Life-Sustaining Treatment (POLST) program in the U.S. (www.polst.org). The My Preferences form aligns with topics in the conversation guides and consists of open sections regarding ‘Living well’, ‘Worries and fears’, ‘Beliefs’, and ‘Hopes’, and a structured section in which patients can indicate their preferences regarding Cardio-Pulmonary Resuscitation (CPR), goals of future care, and final place of care as well as other preferences.  3. INFORMATION LEAFLETS  The facilitators provide leaflets with information regarding ACP and the role of the Personal Representative (PR) to all participants. Where relevant, facilitators also provide leaflets about resuscitation, artificial ventilation and/or artificial feeding. The content of these leaflets was informed by the original RC patient educational materials.  **Fidelity**  For each facilitator, fidelity assessments were conducted twice. In these assessments, the ACTION RC teachers evaluated to what extent the facilitators adhered to the ACTION RC ACP intervention by assessing:   1. To what extent the content of the facilitator’s conversation with patient and, when present, the PR, was in accordance with the ACP Conversation Guide; 2. The facilitator’s general interview (communication) skills; 3. The overall quality of the ACP conversation. |
| --- |
